# Insulinoma in pregnancy: a case report and literature review of diagnostic challenges, multidisciplinary management, and therapeutic dilemmas

**DOI:** 10.3389/fendo.2026.1859250

**Published:** 2026-06-03

**Authors:** Abdullah Faiz Zaihan, Shairyzah Ahmad Hisham, Srinivas Siwalinggam, Noor Hayatul Al Akmal Noralam, Kaeshaelya Thiruchelvam, Chia Siang Kow

**Affiliations:** 1 Department of Pharmacy, Shah Alam Hospital, Shah Alam, Selangor, Malaysia; 2 School of Pharmacy, University of Nottingham Malaysia, Semenyih, Selangor, Malaysia; 3 Department of Radiology, Shah Alam Hospital, Shah Alam, Selangor, Malaysia; 4 Department of Radiology, Sultan Abdul Aziz Shah Hospital, Serdang, Selangor, Malaysia; 5 School of Pharmacy, IMU University, Kuala Lumpur, Malaysia; 6 School of Applied Sciences, University of Huddersfield, Huddersfield, United Kingdom

**Keywords:** endogenous hyperinsulinism, hypoglycaemia, insulinoma, neuroendocrine tumors, pregnancy

## Abstract

**Background:**

Insulinoma during pregnancy is exceedingly rare, with fewer than 30 cases reported worldwide. Diagnosis is often delayed due to overlapping physiological and gestational symptoms, posing significant risks to both mother and fetus.

**Case:**

We report a case of a 36-year-old gravida 4 para 2 + 1 woman presenting at 7 weeks’ gestation with recurrent symptomatic hypoglycaemia. She re-presented at 12 weeks’ gestation with persistent neuroglycopenic symptoms and weight loss. Biochemical evaluation demonstrated inappropriately non-suppressed insulin and C-peptide levels during hypoglycaemia, raising suspicion for endogenous hyperinsulinaemia. Ultrasound was non-diagnostic while magnetic resonance imaging identified a 2.3 x 2.5 cm pancreatic lesion consistent with insulinoma. A multidisciplinary team (MDT) approach involving Endocrinology, Obstetrics & Gynecology (Maternal-Fetal Medicine), Hepatobiliary Surgery, and Radiology guided the management. Due to the tumor size and procedural risks during pregnancy, definitive intervention was deferred while medical therapy with octreotide was initiated, achieving partial glycaemic control. The pregnancy was complicated by fetal growth restriction, necessitating delivery at 34 weeks. Postpartum, she underwent endoscopic ultrasound-guided radiofrequency ablation (EUS-RFA), requiring two sessions before achieving glycaemic stabilization.

**Conclusion:**

This case highlights the diagnostic challenges of insulinoma in pregnancy and underscores the importance of MDT-guided individualized management. It also illustrates the limitations of EUS-RFA and medical therapy, particularly in larger tumors, and raises important considerations regarding fetal outcomes.

## Introduction

Insulinomas are the most common functional pancreatic neuroendocrine tumors, yet they remain rare, with an estimated incidence of approximately 1–4 cases per million person-years (1–3). The diagnosis of insulinoma during pregnancy is exceptionally rare, with fewer than 30 cases reported in the literature when excluding postpartum presentations (4, 5).

Diagnosis in pregnancy is particularly challenging. Classical features such as Whipple’s triad may be obscured by overlapping gestational symptoms including nausea, vomiting, fatigue, and dizziness, which are commonly attributed to early pregnancy or hyperemesis gravidarum. In addition, physiological changes in glucose metabolism during early pregnancy, characterized by increased insulin sensitivity and lower fasting glucose levels may further mask or exacerbate hypoglycaemia, resulting in delayed recognition (4–7).

Management of insulinoma during pregnancy presents a significant clinical dilemma. Treatment strategies must carefully balance the risks of recurrent maternal hypoglycaemia against potential fetal harm. Medical therapies such as diazoxide and somatostatin analogues carry concerns regarding teratogenicity and fetal growth restriction (8, 9), while surgical intervention introduces risks of miscarriage, preterm labor, and maternal morbidity (4). Furthermore, evidence guiding the role of minimally invasive approaches, such as endoscopic radiofrequency ablation, remains limited in this special population (10).

Given the rarity of this condition and the absence of standardized management guidelines, we report a case of insulinoma diagnosed in the first trimester, highlighting diagnostic pitfalls, multidisciplinary decision-making, and therapeutic complexities. To further contextualize our findings, we also performed a focused review of the literature to summarize reported presentations, management strategies, and maternal and fetal outcomes.

The focused literature review was conducted using PubMed/MEDLINE and Scopus from database inception to April 2026. Search terms included combinations of “insulinoma,” “pregnancy,” “pregnant,” “gestation,” “hypoglycaemia,” “hypoglycemia,” “hyperinsulinaemia,” and “pancreatic neuroendocrine tumor.” Additional articles were identified through reference list screening of relevant reports and reviews. Eligible reports included cases of insulinoma diagnosed during pregnancy or presenting with symptoms during pregnancy. Postpartum-only diagnoses and cases diagnosed before conception were excluded unless there was clear evidence of symptom onset or diagnostic evaluation during pregnancy. Extracted data included maternal age, gestational timing, biochemical profile, tumor localization, imaging modality, treatment approach, and maternal and fetal outcomes.

## Case

A 36-year-old woman (gravida 4, para 2, with one prior miscarriage) with no known medical history presented to the Emergency Department at 7 weeks’ gestation with a one-week history of dizziness, presyncope, lethargy, and vomiting. On arrival, her capillary blood glucose (CBG) was 2.5 mmol/L. She was treated with intravenous dextrose and oral glucose, resulting in prompt symptom resolution. Urine analysis demonstrated leukocyturia, and she was treated for a urinary tract infection. She was subsequently discharged with a diagnosis of hypoglycaemia secondary to poor oral intake.

At 12 weeks and 1 day of gestation, she re-presented with recurrent lethargy, severe vomiting, diaphoresis, and shivering. Her CBG was 3.0 mmol/L. She had experienced significant weight loss, from 67.5 kg at 9 weeks’ gestation to 61 kg. Further history revealed recurrent early-morning episodes of diaphoresis and giddiness predating pregnancy, occurring approximately twice monthly and associated with difficulty awakening. These episodes had worsened following conception. Although hypoglycaemic episodes had previously been documented in a primary care setting, no further evaluation had been undertaken.

She was admitted for investigation of recurrent hypoglycaemia. Biochemical evaluation during a hypoglycaemic episode demonstrated findings consistent with endogenous hyperinsulinaemia. Despite a low plasma glucose level, she exhibited inappropriately non-suppressed insulin (82.9 pmol/L; Normal: 17.8 – 173.0 pmol/L) and markedly elevated C-peptide levels (2389 pmol/L; Normal: 366.6 – 1466.6 pmol/L). This strongly supports an endogenous source of hyperinsulinaemia, effectively excluding exogenous insulin administration (11–13). Alternative causes of hypoglycaemia such as adrenal insufficiency were unlikely given preserved cortisol levels (451 nmol/L; Normal: 145.4 - 619.4 nmol/L), while the low Insulin-like Growth Factor 1 (56.7 ng/mL; Normal: 73.1 – 243.0 ng/mL) argues against non-islet cell tumor hypoglycaemia.

Ultrasound of the hepatobiliary system did not identify a pancreatic lesion, consistent with the limited sensitivity of ultrasound for insulinoma detection (9%–64%) (3). This limitation is related to the retroperitoneal location of the pancreas and interference from bowel gas and adipose tissue, which may be further exacerbated during pregnancy (14, 15). Magnetic resonance imaging (MRI) subsequently demonstrated a well-defined lesion measuring 2.3 × 2.5 cm located between the head and tail of the pancreas (**Figure 1**), with features suggestive of insulinoma.

**Figure 1:**
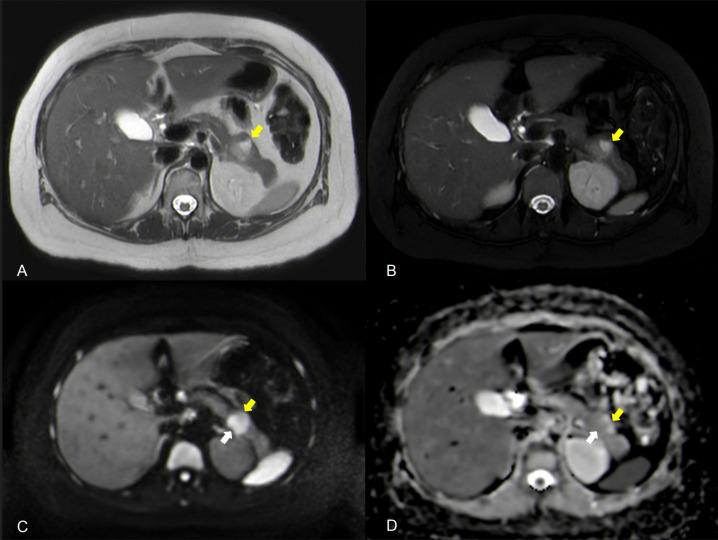
Selected axial MRI images of the upper abdomen demonstrate a well-defined, lobulated lesion in the junction of body-tail of pancreas. The lesion appears markedly hyperintense on T2-weighted imaging [**(A)**, yellow arrow] and remains hyperintense on T2-weighted fat-saturated sequences **(B)**, indicating lack of signal suppression and suggesting a non-fat-containing component. Diffusion-weighted imaging **(C)** reveals focal areas of hyperintensity at the posterior aspect of the lesion, with corresponding low signal on the ADC map **(D)** (white arrow), consistent with true diffusion restriction. These findings are suggestive of increased cellularity within the lesion.

A comprehensive multidisciplinary team comprising endocrinology, maternal-fetal medicine, hepatobiliary surgery, and radiology convened to determine the optimal management strategy. Surgical resection and endoscopic ultrasound-guided radiofrequency ablation (EUS-RFA) were considered. However, given the tumor size (>2 cm) and potential high risks of intervention during pregnancy, a decision was made to pursue conservative management. Diazoxide was avoided due to potential fetal risks. Octreotide therapy was initiated instead, with the aim for a late-preterm delivery (≥34 weeks) to facilitate post-partum surgical or endoscopic intervention.

Following initiation of octreotide, glycaemic control improved, with CBG levels stabilizing between 4.0 – 7.0 mmol/L. She required prolonged inpatient monitoring and was discharged at 19 weeks’ gestation after a 48-day hospitalization. She remained under close outpatient follow-up.

At 34 weeks’ gestation, an elective cesarean delivery was performed. A female infant weighing 1.21 kg was delivered, consistent with stage 1 fetal growth restriction. During this peripartum period, she experienced recurrent severe hypoglycaemia (CBG 2.0 – 3.5 mmol/L) despite escalation of therapy with the addition of diazoxide.

Definitive management was undertaken with Hepatobiliary and Gastroenterology team proceeded with EUS-RFA. EUS confirmed a 2.5 cm hypoechoic lesion in the pancreatic body-tail region, and RFA was performed at 50 watts. Despite the initial RFA, she continued to experience frequent hypoglycaemia, requiring maximal optimization of octreotide and diazoxide. A repeat EUS-RFA was performed two weeks later. A 3.0 cm hypoechoic area was visualized, and RFA was performed utilizing 5 passes, successfully ablating the lesion.

Following the second intervention, she achieved complete clinical and biochemical resolution. Her CBG stabilized between 4.0 – 7.0 mmol/L after discontinuation of medical therapy, and she was discharged in a stable condition.

## Discussion

Insulinoma in pregnancy is an exceptionally rare condition, with fewer than 30 cases reported in the literature when postpartum diagnoses are excluded (4, 5). A synthesis of previously reported cases (**Table 1**) demonstrates consistent patterns in clinical presentation, diagnostic challenges, and management strategies, which provide important context for the present case.

**Table 1:** Characteristics of reported cases of insulinoma in pregnancy identified in the literature review, including timing of onset, biochemical profile, imaging findings, and treatment strategies.

Case	Year	Age	Timing of onset	Nadir glucose (mmol/L)	Biochemical profile	Tumor localization	Key imaging insights	Treatment & timing
1 (16)	1977	21	First trimester	2.0	Markedly elevated insulin (1861 pmol/L)	Pancreatic body (2 cm)	Splenic angiography localized lesion during pregnancy	Open surgical resection at 12 weeks gestation
2 (17)	1983	33	First trimester (week 7)	1.5	Elevated insulin (208–278 pmol/L) with detectable proinsulin	Pancreatic head (2 cm)	Ultrasound and CT localized lesion; angiography supportive	Laparotomy at 17 weeks gestation
3 (18)	1984	19	First trimester	1.7	Elevated insulin (399 pmol/L) with detectable C-peptide	Pancreatic tail (1.8 × 2.6 cm)	CT and angiography identified lesion	Surgical resection following pregnancy termination
4 (19)	1985	24	First trimester (week 10)	1.2	Elevated insulin (201 pmol/L) with preserved C-peptide	Pancreatic body	CT non-diagnostic; angiography localized lesion postpartum	Surgical resection after delivery
5 (20)	1986	24	First trimester (week 6)	0.6	Markedly elevated insulin (1007 pmol/L)	Not localized during pregnancy	Ultrasound, CT, and angiography non-diagnostic	Surgical resection postpartum
6 (21)	1988	37	Third trimester (week 35)	1.1	Elevated insulin (632 pmol/L)	Multiple malignant pancreatic lesions	Imaging non-diagnostic during pregnancy	Surgical intervention after delivery
7 (22)	1988	41	First trimester	1.9	Elevated insulin (438 pmol/L)	Not localized initially	CT and angiography non-diagnostic	Surgical resection postpartum
8 (23)	1990	26	Second trimester (week 16)	1.0	Elevated insulin (104 pmol/L) with elevated C-peptide	Pancreatic tail (2 cm)	Angiography localized lesion postpartum	Surgical resection postpartum
9 (24)	1991	25	First trimester	1.9	Elevated insulin (368 pmol/L) with low-normal C-peptide	Not localized during pregnancy	Ultrasound non-diagnostic	Surgical resection during pregnancy
10 (25)	1992	30	Second trimester (week 16)	0.7	Elevated insulin (184 pmol/L)	Not reported	Imaging not specified	No definitive treatment
11 (26)	1992	24	First trimester	1.3	Mildly elevated insulin (54 pmol/L)	Pancreatic tail	Ultrasound non-diagnostic; MRI later localized lesion	Exploratory surgery in second trimester; definitive resection postpartum
12 (27)	1994	26	Second trimester (week 16)	1.7	Elevated insulin (493 pmol/L) with elevated C-peptide	Pancreatic tail (1 cm)	CT localized lesion postpartum	Surgical resection postpartum
13 (28)	1994	25	First trimester (week 6)	1.9	Elevated insulin (188 pmol/L)	Not localized during pregnancy	Ultrasound non-diagnostic; lesion later identified	Surgical resection at 17 weeks gestation
15 (5)	2002	26	First trimester (week 6)	1.3	Elevated insulin (910 pmol/L) with low C-peptide	Pancreatic tail	MRI and CT localized lesion postpartum	Surgical resection postpartum
19 (29)	2008	22	First trimester (month 2)	2.3	Markedly elevated insulin (477 pmol/L)	Pancreatic tail (~2.5 cm cystic lesion)	MRI localized lesion; EUS confirmed diagnosis	Laparoscopic resection postpartum
20 (30)	2008	29	Third trimester (week 35)	0.9	Elevated insulin (51 pmol/L), C-peptide 0.76 nmol/L, and elevated proinsulin	Mixed cystic and solid pancreatic lesions	Imaging non-diagnostic during pregnancy; intraoperative ultrasound identified lesion	Surgical resection postpartum
22 (31)	2012	29	Second trimester (week 17)	1.6	Not reported	Metastatic disease involving liver	Ultrasound and MRI demonstrated hepatic lesions during pregnancy	Medical therapy (everolimus) postpartum
25 (32)	2017	36	Second trimester (week 17)	2.7	Elevated insulin (29.5 pmol/L), C-peptide (0.54 nmol/L), and proinsulin	Pancreatic tail (~1.8 × 2.2 cm)	EUS and MRI localized lesion during pregnancy	Surgical resection at 21 weeks gestation
28 (33)	2019	31	First trimester (week 10)	1.1	Markedly elevated insulin (~480 pmol/L) with elevated C-peptide (~0.64 nmol/L)	Pancreatic head–neck (~11 mm)	Ultrasound and MRI non-diagnostic; EUS localized tumor	Open surgical enucleation at 18 weeks gestation
27 (34)	2020	30	First trimester (week 13)	1.4	Elevated insulin (79.2 pmol/L) with detectable C-peptide	Pancreatic body (~2.0 × 1.6 cm)	MRI localized lesion during pregnancy	Robotic-assisted enucleation at 18 weeks gestation
29 (6)	2023	34	First trimester (week 10)	1.7	Elevated proinsulin (197.7–380 pmol/L) with detectable C-peptide	Pancreatic body (6 × 10 mm)	Ultrasound and MRI non-diagnostic; EUS localized lesion	Laparoscopic enucleation at 15.5 weeks gestation
30 (35)	2023	28	Second trimester (week 18)	1.5–2.5	Elevated insulin (55 pmol/L), C-peptide (0.8 nmol/L), and proinsulin (53 pmol/L)	Multifocal pancreatic lesions (MEN1), largest 29 mm in tail	MRI demonstrated multiple lesions	Conservative management during pregnancy; distal pancreatectomy postpartum
32 (36)	2025	36	Second trimester (week 24)	2.8	Elevated insulin (~124 pmol/L) with elevated C-peptide (~0.97 nmol/L)	Pancreatic head (10 × 8 mm)	MRI localized lesion; ultrasound non-diagnostic	Dietary management during pregnancy; laparoscopic enucleation postpartum
Our case	2026	36	First trimester (weeks 6–12)	2.5–3.0	Elevated insulin (82.9 pmol/L) with markedly elevated C-peptide (2.39 nmol/L)	Large lesion (~2.3–2.5 cm) in pancreatic body–tail region	MRI localized lesion; ultrasound non-diagnostic	Octreotide during pregnancy followed by EUS-guided radiofrequency ablation ×2 postpartum

The timing and nature of symptom onset in our patient are consistent with prior reports. Most reported cases present during the first trimester or early second trimester, although postpartum presentations are also well described. In several reported cases, patients described intermittent hypoglycaemic symptoms prior to conception that were either mild or misattributed, followed by worsening during early gestation. A similar trajectory was observed in our patient, whose recurrent early-morning hypoglycaemic symptoms predated pregnancy but were not investigated. This highlights a common diagnostic pitfall, where early symptoms are overlooked or attributed to benign causes.

Diagnostic delay is a recurring feature across reported cases. As summarized in **Table 1**, initial misdiagnosis is frequent, with symptoms often attributed to hyperemesis gravidarum, poor oral intake, or other common gestational conditions (4, 6). Our patient was initially managed for hypoglycaemia secondary to poor oral intake, reflecting this pattern. Despite this, biochemical confirmation remains a cornerstone of diagnosis. Where reported, biochemical findings consistently demonstrate inappropriately elevated insulin and C-peptide levels during hypoglycaemia. The biochemical findings in our case are therefore in keeping with previously reported cases, underscoring the importance of early laboratory evaluation in patients with recurrent or unexplained hypoglycaemia.

β-hydroxybutyrate was not available in the clinical records. Although suppressed ketogenesis during hypoglycaemia would have provided additional supportive evidence for endogenous hyperinsulinaemia, the diagnosis was strongly supported by documented hypoglycaemia, inappropriately non-suppressed insulin, markedly elevated C-peptide, MRI localization of a pancreatic lesion, and subsequent biochemical resolution after tumor-directed therapy. There was no known family history of endocrine neoplasia or inherited metabolic disease. Alternative inherited or hormonal causes were considered unlikely given the adult onset, absence of childhood hypoglycaemia, biochemical evidence of endogenous hyperinsulinaemia, radiological localization of a pancreatic lesion, and resolution after tumor-directed therapy.

Imaging findings in our case also parallel those described in the literature. Ultrasound, although commonly used as an initial modality due to its safety profile, frequently fails to detect insulinomas because of its limited sensitivity (3, 14, 15). MRI has emerged as the preferred modality during pregnancy due to its higher diagnostic yield and acceptable safety profile. In our patient, ultrasound was non-diagnostic, whereas MRI successfully localized a pancreatic lesion, consistent with patterns observed in prior reports.

The most important point of comparison lies in management strategies. As outlined in **Table 1**, three principal approaches have been described: medical therapy during pregnancy, surgical resection during the second trimester, and delayed definitive treatment postpartum. The choice of strategy is individualized and depends on tumor characteristics, gestational age, and severity of hypoglycaemia.

Medical therapy is frequently employed as a temporizing measure, particularly in early pregnancy. Octreotide has been the most commonly reported agent, with variable efficacy. In several reported cases, partial glycaemic control was achieved, although breakthrough hypoglycaemia remained a concern. Our case is consistent with this pattern, as octreotide improved glycaemic stability but did not completely prevent recurrent hypoglycaemia, particularly during the peripartum period. In addition, fetal growth restriction has been reported in some cases managed conservatively or with prolonged medical therapy. However, fetal growth restriction in the present case should be interpreted as multifactorial rather than attributed directly to octreotide or conservative management. Potential contributors include significant maternal weight loss, reduced nutritional intake during early pregnancy, recurrent hypoglycaemia, and possible placental insufficiency. Therefore, the fetal growth restriction observed in our patient is best interpreted as an important clinical association that is aligned with previously reported outcomes, rather than as evidence of a direct treatment-related effect.

Surgical resection remains the definitive treatment for insulinoma and has been successfully performed during the second trimester in selected cases, with favorable maternal and fetal outcomes. However, as demonstrated in **Table 1**, surgery is often undertaken in patients with well-localized tumors or in those with severe or refractory hypoglycaemia. In contrast, our patient had a relatively large lesion (>2 cm) in the pancreatic body-tail region, which increased the complexity and risk of surgical intervention. This likely contributed to the decision to defer surgery, consistent with cases in which operative risk was considered high.

Postpartum definitive treatment options include surgical resection and minimally invasive endoscopic ablation. Although surgery remains the standard when complete tumor removal and histological confirmation are required, EUS-RFA was selected in this case after multidisciplinary discussion because the lesion was localized, endoscopically accessible, and suitable for a less invasive approach. This avoided major pancreatic surgery and potentially reduced perioperative morbidity. However, the need for two ablation sessions highlights a limitation of EUS-RFA, particularly for larger lesions.

Minimally invasive approaches, such as EUS-RFA, are increasingly used in non-pregnant populations but remain poorly described in pregnancy. Our case contributes to this limited body of evidence. Notably, while most reported cases undergoing definitive treatment achieve resolution following a single intervention, our patient required two sessions of radiofrequency ablation to achieve biochemical remission. This suggests that larger lesions may be less amenable to single-session ablation, an observation that has not been consistently reported and may have implications for procedural planning.

Maternal outcomes across reported cases are generally favorable with appropriate management. However, fetal outcomes are more variable. As summarized in **Table 1**, fetal growth restriction and preterm delivery are among the reported complications, particularly in cases managed conservatively or with prolonged medical therapy. Our case is consistent with these findings, with the development of fetal growth restriction necessitating delivery at 34 weeks’ gestation.

An important concept emerging from comparison with the literature is the timing of definitive treatment. Two broad management pathways can be identified: immediate intervention during pregnancy and delayed treatment postpartum. Our case supports a staged approach, whereby maternal glycaemic control is achieved during pregnancy using medical therapy, followed by definitive intervention after delivery. This strategy may be particularly appropriate in patients with stable disease or when procedural risks are considered high.

Importantly, our case demonstrates several features that distinguish it from previously reported cases. First, the patient presented early in the first trimester with symptoms that clearly predated pregnancy, yet remained uninvestigated until gestation, further emphasizing the extent of diagnostic delay that can occur. Second, although octreotide is commonly used, our case highlights its limitations in achieving sustained glycaemic control, particularly during the peripartum period despite escalation of therapy. Third, definitive treatment required two sessions of endoscopic ultrasound-guided radiofrequency ablation, suggesting that larger tumors may require staged or repeated interventions. Finally, this case illustrates the successful application of a staged management strategy, supporting delayed postpartum intervention in selected patients.

Given the rarity of insulinoma in pregnancy and the absence of standardized management guidelines, a multidisciplinary approach is essential. Close collaboration between endocrinology, maternal-fetal medicine, surgery, and radiology allows for individualized decision-making and optimization of maternal and fetal outcomes.

This report has several limitations. The available evidence is limited to case reports and small case series, resulting in heterogeneity in reporting and management strategies. In addition, comparisons across cases are descriptive and not based on standardized outcome measures. Histological confirmation, Ki-67 index, and tumor grading were not available because definitive treatment was performed using EUS-guided radiofrequency ablation rather than surgical resection. This limits pathological characterization of the lesion. Biochemical profile, imaging findings, and clinical response to tumor-directed therapy strongly supported the diagnosis of insulinoma. Nevertheless, by integrating our case with a focused synthesis of previously reported cases, we provide clinically relevant insights into the diagnosis and management of insulinoma in pregnancy.

In conclusion, insulinoma in pregnancy is a rare but often under-recognized cause of hypoglycaemia, with diagnosis frequently delayed by nonspecific symptoms and limited imaging sensitivity. Biochemical confirmation remains essential, while MRI and EUS improve tumor localization. Management should be individualized, balancing maternal stability and fetal safety. Our case supports a staged approach, with medical stabilization during pregnancy followed by definitive postpartum intervention, and highlights that larger tumors may require repeated treatment. Early recognition and multidisciplinary care are critical to optimizing maternal and fetal outcomes.

## Data Availability

The clinical data supporting the findings of this case report are included within the article. Additional patient-level information is not publicly available to protect patient confidentiality and privacy. Further anonymised information may be made available from the corresponding author upon reasonable request, subject to institutional and ethical approval. Data used for the literature review were obtained from previously published articles cited in the reference list.

## References

[1] de Herder WW Hofland J . Insulinoma. In: Feingold KR Adler RA Ahmed SF Anawalt B Blackman MR Chrousos G , editors.Endotext. South Dartmouth (MA): MDText.com, Inc (2023). Internet.

[2] Tsang YP Lang BH Shek TW . Assessing the short- and long-term outcomes after resection of benign insulinoma. Anz J Surg. (2016) 86:706–10. doi: 10.1111/ans.12891. PMID: 25345822

[3] Okabayashi T Shima Y Sumiyoshi T Kozuki A Ito S Ogawa Y . Diagnosis and management of insulinoma. World J Gastroenterol. (2013) 19:829–37. doi: 10.3748/wjg.v19.i6.829. PMID: 23430217 PMC3574879

[4] Dobrindt EM Mogl M Goretzki PE Pratschke J Dukaczewska AK . Insulinoma in pregnancy: systematic review of the literature. Rare Tumors. (2021) 13:2036361320986647. doi: 10.1177/2036361320986647 33613925 PMC7874339

[5] Takacs CA Krivak TC Napolitano PG . Insulinoma in pregnancy: a case report and review of the literature. Obstet Gynecol Surv. (2002) 57:229–35. doi: 10.1097/00006254-200204000-00022. PMID: 11961480

[6] Diaz-Sangines BP Gonzalez-Cofrades J Vazquez-Camacho EE Malfavon-Farias M Garcia-Lima L . Insulinoma management in a pregnant woman: a case report. Cureus. (2023) 15:e34239. doi: 10.7759/cureus.34239. PMID: 36852368 PMC9964046

[7] Besemer B Müssig K . Insulinoma in pregnancy. Exp Clin Endocrinol Diabetes. (2010) 118:9–18. doi: 10.1055/s-0029-1202272. PMID: 19373751

[8] Andrade J . Diazoxide. In: Encyclopedia of toxicology, vol. 3. Amsterdam: Elsevier (2024). p. 597–600. doi: 10.1016/B978-0-12-824315-2.00615-1

[9] Geilswijk M Andersen LL Frost M Brusgaard K Beck-Nielsen H Frederiksen AL . Octreotide therapy and restricted fetal growth: pregnancy in familial hyperinsulinemic hypoglycemia. Endocrinol Diabetes Metab Case Rep. (2017) 2017:16–0126. doi: 10.1530/EDM-16-0126. PMID: 28458896 PMC5404468

[10] Imperatore N de Nucci G Mandelli ED de Leone A Zito FP Lombardi G . Endoscopic ultrasound-guided radiofrequency ablation of pancreatic neuroendocrine tumors: a systematic review of the literature. Endosc Int Open. (2020) 8:E1759–64. doi: 10.1055/a-1261-9605. PMID: 33269308 PMC7671767

[11] Blocher NC . C-peptide Medscape (2025) (Accessed April 12, 2026).

[12] Cryer PE . Hypoglycemia in diabetes: pathophysiological mechanisms and diurnal variation. Prog Brain Res (2006) 153:361–365. doi: 10.1016/S0079-6123(06)53021-3 16876586

[13] Vezzosi D Bennet A Fauvel J Caron P . Insulin, C-peptide and proinsulin for the biochemical diagnosis of endogenous hyperinsulinism. Eur J Endocrinol. (2007) 157:75–83. doi: 10.1530/eje-07-0109. PMID: 17609405

[14] Ali ZA . Insulinoma workup: approach considerations, laboratory studies, imaging studies Medscape (2024) (Accessed April 4, 2026).

[15] Zhao K Patel N Kulkarni K Gross JS Taslakian B . Essentials of insulinoma localization with selective arterial calcium stimulation and hepatic venous sampling. J Clin Med. (2020) 9:3091. doi: 10.3390/jcm9103091. PMID: 32992761 PMC7601191

[16] Rubens R Carlier A Thiery M Vermeulen A . Pregnancy complicated by insulinoma: case report. Br J Obstet Gynaecol. (1977) 84:543–7. doi: 10.1111/j.1471-0528.1977.tb12643.x. PMID: 199230

[17] Wilson JD Hugh TB . Pregnancy complicated by an insulinoma. Aust N Z J Obstet Gynaecol. (1983) 23:176–9. doi: 10.1111/j.1479-828x.1983.tb00570.x. PMID: 6316901

[18] Osei K Kramer DS Malarkey WB Falko JM . Pregnancy complicated by insulinoma. Am J Med Sci. (1984) 288:133–5. doi: 10.1097/00000441-198410000-00008. PMID: 6091457

[19] Shaw DL Bernene JL Williams JW North CQ Palestrant AM . Insulinoma in pregnancy. Ariz Med. (1985) 42:406–8. 2994605

[20] Galun E Ben-Yehuda A Berlatzki J Ben-Chetrit E Gross DJ . Insulinoma complicating pregnancy: case report and review of the literature. Am J Obstet Gynecol. (1986) 155:64–5. doi: 10.1016/0002-9378(86)90079-7. PMID: 3014882

[21] Friedman E Moses B Engelberg S Levran D Lieberman P . Malignant insulinoma with hepatic failure complicating pregnancy. South Med J. (1988) 81:86–8. doi: 10.1097/00007611-198801000-00019. PMID: 2827322

[22] Hale PJ Hale JF Nattrass M . Insulinoma and pregnancy: case report. Br J Obstet Gynaecol. (1988) 95:514–7. doi: 10.1111/j.1471-0528.1988.tb12808.x. PMID: 2840943

[23] Smythe AR McFarland KF Yousufuddin M Meredith WD . Multiple endocrine adenomatosis type I in pregnancy. Am J Obstet Gynecol. (1990) 163:1037–8. doi: 10.1016/0002-9378(90)91121-r. PMID: 1976295

[24] Liberman C Valenzuela MA Hernandez F Miranda C Salazar V Castillo J . Insulinoma and pregnancy: clinical case. Rev Med Chile. (1991) 119:564–6. 1844297

[25] Akanji AO George AO Olasode BJ Osotimehin BO . Insulinoma in pregnancy presenting as a seizure disorder: a case report. East Afr Med J. (1992) 69:117–9. 1505386

[26] Atala C Tapia M . Insulinoma and pregnancy: a clinical case. Rev Chil Obstet Ginecol. (1992) 57:437–9. 1364570

[27] Auinger M Dudczak R Fasching W Leodolter S Feinbock C Irsigler K . Detection of an insulinoma in pregnancy: a rare cause of hypoglycemia. Wien Klin Wochenschr. (1994) 106:426–9. 8091767

[28] Bardet S Mahot P Deumier B Le Neel JC Krempf M Charbonnel B . Discovery of an insulinoma during the first trimester of pregnancy. Presse Med. (1994) 23:285–7. 8208679

[29] Diaz AG Herrera J Lopez M Puchulu FM Ferraina P Bruno OD . Insulinoma associated with pregnancy. Fertil Steril. (2008) 90:199:e1–e4. doi: 10.1016/j.fertnstert.2007.06.092. PMID: 17980876

[30] Christiansen E Vestergaard H . Insulinoma in a third-trimester pregnant woman combined with pre-eclampsia: a case report and review of the diagnostic strategies. Gynecol Endocrinol. (2008) 24:417–22. doi: 10.1080/09513590802210931. PMID: 18645715

[31] Mannelli L Yeh MM Wang CL . A pregnant patient with hypoglycemia. Gastroenterology. (2012) 143:e3–4. doi: 10.1053/j.gastro.2012.03.051. PMID: 22921669

[32] Tomazic M Janez A Ravnik Oblak M . Hypoglycemia identified by a continuous glucose monitoring system in a second-trimester pregnant woman with insulinoma: a case report. J Med Case Rep. (2017) 11:117. doi: 10.1186/s13256-017-1265-8. PMID: 28427440 PMC5399342

[33] Taweerutchana V Choksakunwong S Lerwattrakarn A Chalermwai WV Intralawan T Lertwattanarak R . Pioneering robotic-assisted surgery for insulinoma during pregnancy: the first case report and literature review. Heliyon. (2024) 10:e34239. doi: 10.1016/j.heliyon.2024.e34239. PMID: 39100462 PMC11296031

[34] de Albuquerque Neto CC da Silva Lira N Albuquerque MARC Santa-Cruz F de França M Vasconcelos L Ferraz ÁAB . Surgical resection of pancreatic insulinoma during pregnancy: case report and literature review. Int J Surg Case Rep. (2019) 61:119–22. doi: 10.1016/j.ijscr.2019.07.019. PMID: 31357102 PMC6664231

[35] Wood AJ Kasireddy V Chitturi S Walsh JP . Insulinoma presenting with postprandial hypoglycemia in a pregnant woman with MEN-1. JCEM Case Rep. (2022) 1:luac015. doi: 10.1210/jcemcr/luac015. PMID: 37908256 PMC10578399

[36] Aksoy SB Özsoy M Erman H Gökkaya N Küçük HF Aydıner Ö . Insulinoma in pregnancy: is diet alone enough to treat insulinoma? Endokrynol Pol. (2025) 76:577–8 41457635 10.5603/ep.105639

